# Supplementary material for: Mechanisms of virus dissemination in bone marrow of HIV-1–infected humanized BLT mice
Source: eLife. 2019 Oct 28;8:e46916. doi: 10.7554/eLife.46916 (PMC6839903; doi:10.7554/eLife.46916)
Supplement: Transparent reporting form [file elife-46916-transrepform.pdf]

eLIFE
1st Floor
24 Hills Road
Cambridge CB2 1JP, UK
P 01223 855340
W elifesciences.org
T @elife

eLife's transparent reporting form

We encourage authors to provide detailed information *within their submission* to facilitate the interpretation and replication of experiments. Authors can upload supporting documentation to indicate the use of appropriate reporting guidelines for health-related research (see EQUATOR Network), life science research (see the BioSharing Information Resource), or the ARRIVE guidelines for reporting work involving animal research. Where applicable, authors should refer to any relevant reporting standards documents in this form.

If you have any questions, please consult our Journal Policies and/or contact us: editorial@elifesciences.org.

Sample-size estimation

- You should state whether an appropriate sample size was computed when the study was being designed
- You should state the statistical method of sample size computation and any required assumptions
- If no explicit power analysis was used, you should describe how you decided what sample (replicate) size (number) to use

Please outline where this information can be found within the submission (e.g., sections or figure legends), or explain why this information doesn't apply to your submission:

The bone marrow, liver, thymus humanized mouse model for HIV-1 infection utilized in this study is an expensive and labor intensive model and thus prevented us from utilizing large numbers of replicate animals for our studies. We included one uninfected control animal (day 0) in order to show that steady state immune reconstitution levels were similar to those of the infected animals at the same time point and that no HIV-1 was detectable prior to infection. We included three animals at early time points because one focus of this study was to evaluate at what stage post-infection HIV-1 can be detected in bone marrow and compare that to the levels of HIV-1 in the bloodstream. We additionally included two HIV-1-infected animals at later timepoints to evaluate HIV-1 pathogenesis in bone marrow at times after establishment of infection.

Replicates

- You should report how often each experiment was performed
- You should include a definition of biological versus technical replication
- The data obtained should be provided and sufficient information should be provided to indicate the number of independent biological and/or technical replicates
- If you encountered any outliers, you should describe how these were handled
- Criteria for exclusion/inclusion of data should be clearly stated
- High-throughput sequence data should be uploaded before submission, with a private link for reviewers provided (these are available from both GEO and ArrayExpress)

Please outline where this information can be found within the submission (e.g., sections or figure legends), or explain why this information doesn't apply to your submission:

Figure 1 - Legend mentions 3 replicates (separate regions of tissue) from each time point and includes images from a single of those replicates.

In sum, 5 total animals were infected with HIV-1 and all showed productive infection as biological replicates as compared to uninfected. As mentioned above, these 5 biological replicates could be further subdivided into early time point animals (n=3) and late time point animals (n=2) representing biological replicates. Concerning sample size, we were limited in the number of replicate animals we could generate, infect, and analyze with the methods utilized in our study. As mentioned in the methods section for immunofluorescence image processing "Three volumes were quantified from 1-2 samples for each time point".

Statistical reporting

- Statistical analysis methods should be described and justified
- Raw data should be presented in figures whenever informative to do so (typically when N per group is less than 10)
- For each experiment, you should identify the statistical tests used, exact values of N, definitions of center, methods of multiple test correction, and dispersion and precision measures (e.g., mean, median, SD, SEM, confidence intervals; and, for the major substantive results, a measure of effect size (e.g., Pearson's r, Cohen's d)
- Report exact p-values wherever possible alongside the summary statistics and 95% confidence intervals. These should be reported for all key questions and not only when the p-value is less than 0.05.

Please outline where this information can be found within the submission (e.g., sections or figure legends), or explain why this information doesn't apply to your submission:

Figure 1 E legend - Standard deviations for error bars are described. The focus of this graph is to indicate the large change in density of hCD4+ cells as compared to hCD68+ cells over time.

Figure 1 F legend - Standard deviations for error bars are described. The focus of this graph was to indicate that CD68+ macrophages unexpectedly made a up a large proportion of the p24+ cells in bone marrow

Finally, several of our figures are descriptive and focus on visual results from a limited number of samples analyzed.

(For large datasets, or papers with a very large number of statistical tests, you may upload a single table file with tests, Ns, etc., with reference to sections in the manuscript.)

Group allocation

- Indicate how samples were allocated into experimental groups (in the case of clinical studies, please specify allocation to treatment method); if randomization was used, please also state if restricted randomization was applied
- Indicate if masking was used during group allocation, data collection and/or data analysis

Please outline where this information can be found within the submission (e.g., sections or figure legends), or explain why this information doesn't apply to your submission:

Individual animals exhibiting human immune cell reconstitution were randomly placed into experimental groups/time points and levels of HIV or immune cells in the blood (Figure 1-figure supplement 1) were not assessed until after sacrifice. At the time of sacrifice, it was not known whether productive infection of each individual animal had been established.

Additional data files ("source data")

- We encourage you to upload relevant additional data files, such as numerical data that are represented as a graph in a figure, or as a summary table
- Where provided, these should be in the most useful format, and they can be uploaded as "Source data" files linked to a main figure or table
- Include model definition files including the full list of parameters used
- Include code used for data analysis (e.g., R, MatLab)

eLife Sciences Publications, Ltd is a limited liability non-profit non-stock corporation incorporated in the State of Delaware, USA, with company number 5030732, and is registered in the UK with company number FC030576 and branch number BR015634 at the address 1st Floor, 24 Hills Road, Cambridge CB2 1JP | August 2014

- Avoid stating that data files are "available upon request"

Please indicate the figures or tables for which source data files have been provided:

Figure 1 contains source data files that have been uploaded.

eLife Sciences Publications, Ltd is a limited liability non-profit non-stock corporation incorporated in the State of Delaware, USA, with company number 5030732, and is registered in the UK with company number FC030576 and branch number BR015634 at the address 1st Floor, 24 Hills Road, Cambridge CB2 1JP | August 2014
